# The effects of fixational tremor on the retinal image

**DOI:** 10.1167/19.11.8

**Published:** 2019-09-18

**Authors:** Norick R. Bowers, Alexandra E. Boehm, Austin Roorda

**Affiliations:** nbowers2@berkeley.edu; aeboehm@berkeley.edu; aroorda@berkeley.edu; School of Optometry and Vision Science Graduate Group, University of California–Berkeley, Berkeley, CA, USA; School of Optometry and Vision Science Graduate Group, University of California–Berkeley, Berkeley, CA, USA; School of Optometry and Vision Science Graduate Group, University of California–Berkeley, Berkeley, CA, USA

**Keywords:** *tremor*, *fixational eye motion*, *scanning laser ophthalmoscopy*, *adaptive optics*

## Abstract

The study of fixational eye motion has implications for the neural and computational underpinnings of vision. One component of fixational eye motion is tremor, a high-frequency oscillatory jitter reported to be anywhere from ∼11–60 arcseconds in amplitude. In order to isolate the effects of tremor on the retinal image directly and in the absence of optical blur, high-frequency, high-resolution eye traces were collected in six subjects from videos recorded with an adaptive optics scanning laser ophthalmoscope. Videos were acquired while subjects engaged in an active fixation task where they fixated on a tumbling E stimulus and reported changes in its orientation. Spectral analysis was conducted on periods of ocular drift, with all drifts being concatenated together after removal of saccades from the trace. The resultant amplitude spectra showed a slight deviation from the traditional 1/*f* nature of optical drift in the frequency range of 50–100 Hz, which is indicative of tremor. However, this deviation rarely exceeded 1 arcsecond and the consequent standard deviation of retinal image motion over the tremor band (50–100 Hz) was just over 5 arcseconds. Given such a small amplitude, it is unlikely tremor will contribute in any meaningful way to the visual percept.

## Introduction

### Fixational eye motion

Even during intersaccadic periods of fixation the eye is never still; small eye movements constantly shift the retinal image over the photoreceptor mosaic. These movements can shift a stimulus over dozens of photoreceptors every second. Fixational eye motion has typically been categorized to fall into three main components: (a) Microsaccades, small ballistic movements similar to larger saccades, (b) Ocular drift, a slow Brownian-like movement shifting the gaze only a few arcminutes, and (c) Tremor, a high-frequency oscillatory jitter roughly the size of a foveal cone (Ditchburn & Ginsborg, 1953; Eizenman, Hallett, & Frecker, 1985; Ko, Snodderly, & Poletti, 2016; Ratliff & Riggs, 1950; Rucci & Poletti, 2015). Extensive research has been conducted on the functional and perceptual consequences of microsaccades and drift (Bowers & Poletti, 2017; Burak, Rokni, Meister, & Sompolinsky, 2010; Engbert, 2006; Kagan, Gur, & Snodderly, 2008; Ko, Poletti, & Rucci, 2010; Kuang, Poletti, Victor, & Rucci, 2012; Martinez-Conde, Macknik, Troncoso, & Dyar, 2006; Ratnam, Domdei, Harmening, & Roorda, 2017; Rolfs, 2009; Rucci, Iovin, Poletti, & Santini, 2007), but the perceptual consequences of tremor and its possible functional role are still not fully understood.

### Tremor

Reports of tremor vary widely on the statistical nature and magnitude of this motion. Tremor is generally defined as an increase in eye motion amplitude at high frequencies. The bandwidth of tremor is often reported as falling between 50 Hz and 100 Hz, whereas the magnitude of motion has been found to be as small as 11.1 arcseconds (average standard deviation of eye movements within the tremor band reported by Ko et al., 2016) and as large as 1 arcminute (visual observation of eye traces by Ratliff & Riggs, 1950) and some reports question the existence of tremor at all (Stevenson, Roorda, & Kumar, 2010). Few studies have set out to examine the implications of tremor for vision, largely due to the technical difficulties of accurately measuring tremor with conventional eye trackers. There is some evidence that tremor could contribute to perception by synchronizing retinal ganglion cells (Greschner, Bongard, Rujan, & Ammermüller, 2002) or through stochastic resonance of visual noise (Hennig, Kerscher, Funke, & Wörgötter, 2002).

### Eye tracking

Most reports of tremor stem from the use of high-resolution eye tracking techniques, such as dual-Purkinje image (DPI) eye tracking (Crane & Steele, 1985; Ko et al., 2016), scleral search coils (Houben, Goumans, & Van Der Steen, 2006), reflections from small mirrors placed on contact lenses (Ditchburn & Ginsborg, 1953; Ratliff & Riggs, 1950; Riggs & Schick, 1968; Steinman, Haddad, Skavenski, & Wyman, 1973; Yarbus, 1968), and reflections from the cornea directly (Eizenman et al., 1985). Each of these trackers has the potential spatial and temporal resolution to measure tremor, but each relies on tracking some part of the anterior segment or lens of the eye and inferring the motion on the retina. The current study looks to examine the effects of tremor on the retinal image directly using an adaptive optics scanning laser ophthalmoscope (AOSLO), a relatively novel method of tracking the eye that relies on imaging the retinal surface directly (Stevenson & Roorda, 2005; Vogel, Arathorn, Roorda, & Parker, 2006).

## Methods

### Adaptive optics system

Movies of the retina are obtained through the use of an adaptive optics scanning laser ophthalmoscope (AOSLO; Roorda et al., 2002). In the AOSLO system, a focused point of light is scanned across the retina in a raster pattern to obtain high-resolution movies of the photoreceptor mosaic during fixation. In the most recent version of the system, a supercontinuum light source provides a point source of light that is relayed through the optical path to a fast horizontal resonant scanner (16 kHz) and a slow vertical scanner (30 Hz), which together sweep the point across the retina in a raster pattern, and the deformable mirror (7.2 mm diameter 97 actuator membrane; ALPAO, Montbonnot-Saint-Martin, France), which compensates for the aberrations of the eye. The light reflecting from the retina is relayed and descanned back through the optical path to a custom Shack-Hartmann wavefront sensor, which measures the aberrations, through a confocal pinhole (conjugate to the retinal plane of focus), and then to a photomultiplier tube, which is used to record the scattered light, pixel-by-pixel, to reconstruct an image of the retinal surface. The AOSLO system used is equipped with four wavelength channels: 840, 680, and 543 nm channels are used for imaging and stimulus delivery, and a 940 nm wavelength channel is used for wavefront sensing. In this particular experiment, 840 nm (40–60 microWatts average power) was used for imaging and 543 nm was used to provide a stimulus for fixation (see Experimental design). The vergence of all wavelengths were adjusted in the light delivery arm to compensate for longitudinal chromatic aberration so that all wavelengths were in simultaneous focus on the retina (Grieve, Tiruveedhula, Zhang, & Roorda, 2006; Harmening, Tiruveedhula, Roorda, & Sincich, 2012). Custom software was used to operate the entire AO control system. Measurement and correction were performed over the entire pupil diameter up to a maximum of 7.2 mm. This system generally obtains near diffraction-limited images of the retina with high enough spatial resolution to resolve foveal cones.

### Strip-based eye tracking

The images obtained by the AOSLO system were compiled together in a continuous sequence to create movies of the retina. The movies were acquired at 30 Hz (the frequency of the slow vertical scanner) and were composed of 512 × 512 pixels. The size of the raster on the retina was computed by imaging a calibration grid on a model eye to be 0.9° so that each arcminute is subtended by ∼10 pixels. Eye movement traces were acquired from the movies using an offline algorithm that utilized a strip-based cross correlation technique to obtain eye traces at higher temporal resolution than the frame rate of the system (Stevenson & Roorda, 2005; Vogel et al., 2006). Since each frame was acquired over time, additional temporal information on eye movements, which manifest as unique distortions within each frame, is available beyond the 30 Hz frame rate. The top of each frame occurs earlier in time than the bottom, and by dividing each frame into strips and analyzing the movement in a strip-wise manner, eye motion traces can be acquired with a much higher temporal sampling rate than the frame rate of the movie (30 Hz). The eye motion sampling rate is the frame rate multiplied by the number of strips per frame, so the temporal resolution of the eye motion can be adjusted by increasing or decreasing the number of strips used in the cross-correlation. For the current study, 64 strips were used per frame, giving an eye-motion sampling rate of 1920 Hz. The eye motion correction has been done in real time (Arathorn et al., 2007) and offline (Stevenson et al., 2010). For all analyses in this paper, eye motion computations were done offline after the videos were acquired. Eye motion traces were converted from pixels to units of arcminutes using the scaling described above.

For offline analysis, an oversized composite reference frame is generated for each movie by averaging together and roughly aligning selected frames of the movie. The size of the composite reference is dependent on the extent of eye motion during recording. Each frame of the movie is then divided into 64 strips that are 8 pixels in height and run the entire 512 pixels of the frame width. Each strip is cross-correlated against the reference frame in order to obtain vertical and horizontal offsets of the eye position compared to the reference at that instance. Each strip represents one sample of the eye trace and the strips from each frame are strung together into a continuous sequence to obtain eye traces at a rate of 1920 Hz from the 30 Hz AOSLO movies. This technique can detect eye motion with amplitudes smaller than one arcsecond (Stevenson et al., 2010).

### Eye movement parsing

Once the raw eye traces were acquired, they were parsed using an automatic algorithm. First, erroneous or noisy eye motion traces recorded during blinks or during periods in which the image quality was very poor were identified by labeling frames in the movie in which the average luminance of the total frame fell below a threshold. Second, saccades were identified using a speed threshold, wherein saccade onset was considered the point in which the eye moved above 1.5 °/s and saccade offset was considered the point in which the eye fell below 1.5 °/s. Saccades falling below an amplitude threshold (3 arcminutes) were not considered for analysis. Any consecutive saccadic events that occurred within 15 ms of each other were merged into one event in order to automatically eliminate saccade overshoot. This technique is similar to the one employed by Ko et al. (2016). Drift was identified as all intersaccadic periods of eye motion. Saccade detection was verified by human observers manually to identify any saccades the automatic algorithm may have missed. In the data sets collected here on young healthy eyes with normal fixation (see Experimental design), the automatic algorithm captured most saccades and only a small number had to be manually identified. Whenever a frame contained poor data or a saccade, the entire frame was flagged and not considered in the analysis. The first sample of each drift frame was repositioned to align with the last sample of the previous drift segment to eliminate discontinuous jumps from saccades and blinks. This technique was used because intersaccadic periods of drift are generally small (see statistics in the Results section), which poses a constraint on the resolution of the Fourier analysis. Drifts stitched together in this method were cropped together using only full frames worth of samples (∼33 ms periods), that is, any portion of a drift that began or ended in the middle of a frame was not included. On average, 40 ms were cut from the beginning and end of each drift before stitching them together, although the exact amount varied from drift to drift. This was done in order to better eliminate periodic artifacts at the frame rate of the system arising from torsion or reference frame distortions (see section under heading Reference frame and torsion correction). This method assumes stationarity of intersaccadic drift segments (disregarding any polar bias in drift direction). All of the eye motion traces are available for download in the resources section of this website: roorda.vision.berkeley.edu. Each data file contains two traces (a) a complete eye motion trace with each sample identified as a drift, saccade, blink, or bad data, and (b) the concatenated drift segment that was used for the spectral analysis.

### Reference frame and torsion correction

Although the eye motion traces after parsing produce continuous segments of isolated drift at high sampling rates, the traces still contain motion artifacts caused by distortions in the reference frame as well as torsional eye movements. Torsional eye movements produce a sawtooth waveform that repeats at the frame rate of the system, whereas reference frame distortions present as a short random walk overlaid onto each frame's motion. In the case of the current study, a reference frame distortion will be a random walk constructed of 64 samples (the number of strips/samples per frame) and overlaid onto every set of 64 samples throughout the trace. Fortunately, both of these artifacts are periodic and introduce peaks in the amplitude spectra that are isolated to the frame rate of the system and higher harmonics only; they do not affect the underlying amplitude spectrum anywhere else. In the eye motion traces from the offline-processed videos, reference frame artifacts are largely removed by using multiple frames to generate a composite reference frame (Stevenson et al., 2010). By combining a series of frames, the distortions of the individual frames are averaged out (Bedggood & Metha, 2017). Torsion, however, may change over the course of a video and the periodic sawtooth must be removed from each frame individually. (A full description of the algorithms to measure and remove reference frame distortion and torsional artifacts is part of a paper in progress.) For the purposes of this paper, it is sufficient to state that the sawtooth artifact was measured and removed from the eye motion trace prior to further analysis.

### Eye motion analysis

The amplitude spectra of these drift segments were then analyzed by doing a multitaper spectral analysis on the X and Y eye motion traces expressed in units of amplitude in arcminutes versus time in seconds, originally proposed by Thomson (1982) and more recently reviewed by Babadi and Brown (2014). The analysis was done using the command *pmtm* in the MATLAB Signal Processing Toolbox (MathWorks, Natick, MA). Briefly, the spectral analysis method involves running an fast Fourier transform (FFT) on a motion trace using overlapping and mutually orthogonal tapers (discrete prolate spheroidal sequences). For the current manuscript, the analysis was run on overlapping segments (50% overlap) of 1 s, which comprised 1,920 samples each, allowing for analysis of frequencies sampled in 1-Hz steps up to 960 Hz. In the MATLAB function, the time half-bandwidth product was fixed at 2.5 and the last taper was dropped to maximize spectral concentration ratios in the Slepian sequences. In general, we found that variations in the bin size, amount of overlap, and time half-bandwidth product had a minimal effect on the final output in the frequency range of tremor (50–100 Hz). The square root of the output spectra were taken to convert from power to amplitude in arcmin/Hz in order to better capture the motion on the retina and to facilitate a more direct comparison with previously published results. To compute the actual motion of the retinal image caused by tremor, we employed similar methods used by Ko et al. (2016) wherein we bandpass-filtered the eye motion traces to contain only the motion within the tremor band (50–100 Hz) and computed the standard deviation of the filtered eye motion trace.

### Validations

Two tests were done in order to validate the ability of the AOSLO system to track eye motion. The first validation aimed to test the ability of the entire AOSLO system to record motion. A model eye was attached to a galvanometer and oscillated in a diagonal sinusoidal pattern at four frequencies (4 Hz, 16 Hz, 64 Hz, and 256 Hz) while being recorded with the AOSLO system. To enable analysis of the noise floor of the system, a video was also recorded of the nonmoving model eye. Thirty-second movies were collected at each frequency with a fixed amplitude of 0.45 arcminutes at an angle of ∼26.6° to give horizontal and vertical amplitudes of ∼0.4 and ∼0.2 arcminutes, respectively. The motion traces from these movies were analyzed by averaging together the amplitude spectra of a series of nonoverlapping 1-s segments of the trace. Unlike the eye motion traces, a simple FFT computation was used here since it is more suitable for single-frequency motion traces.

The second validation was a simulation aimed to test the ability of the strip-based cross correlation technique to recover motion from actual AOSLO movies. First, a real AOSLO movie was manipulated digitally to add a distortion that would result from tremor. Specifically, we added motion from a parabolic-weighted band of frequencies ranging from 50–100 Hz with a peak in the amplitude spectrum of 2 arcseconds. Following the manipulation, the movie was analyzed using the strip-based cross correlation technique described above to obtain traces of eye motion at 1920 Hz. The modified AOSLO movie was analyzed using the same multitaper spectral analysis methods described above. The amplitude spectra of the motion trace from the manipulated movie were compared against the amplitude spectra of the motion trace from the original movie. We felt that validation with an actual AOSLO video was important because in a model eye, the luminance and contrast of the image is static and the retina moves in only the direction of the galvanometers. The AOSLO movie, in contrast, contains actual eye motion and has more variable luminance owing to changes in the adaptive optics correction as well as actual changes in reflected intensity from the retina (Pallikaris, Williams, & Hofer, 2003). If our eye motion analysis can recover the frequency and amplitude of high frequency motion that has been added to an actual movie, then we can be confident that the strip-based eye-tracking algorithm would be able to detect real eye motion at these frequencies.

### Experiment design

**Figure 1:**
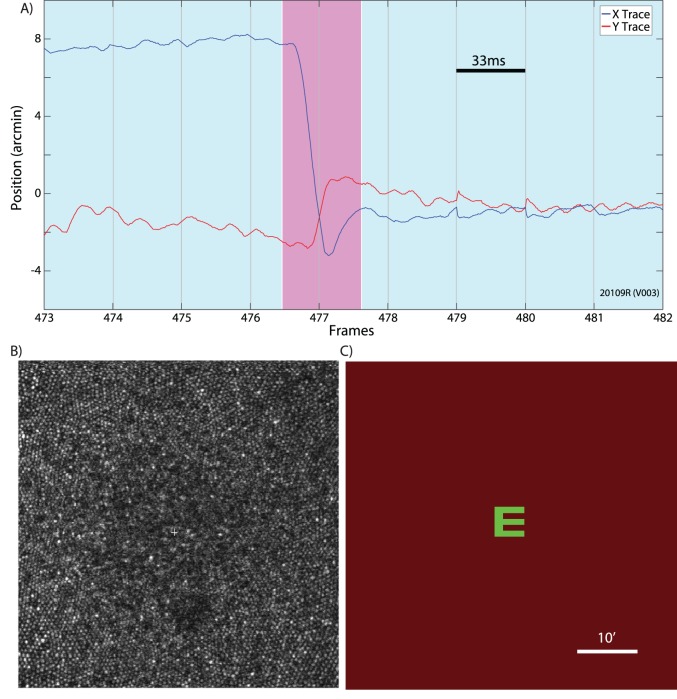
(A) An example of an eye trace taken from an AOSLO movie. A microsaccade (magenta background) is clearly distinguishable from the ocular drift (blue background). Gray vertical gridlines demarcate frame boundaries from the AOSLO movie. Each frame is acquired over 33 ms as indicated by the scale bar. (B) An example of an image/frame from an AOSLO movie. The cone mosaic can be resolved even at the fovea. (C) An example of the AOSLO raster with a green letter E as it would appear to the subject. The small discontinuities in the eye trace at the boundaries between frames 478–479 and 480–481 are likely the result of tracking errors that occur at the edges of the frame. They are infrequent and an example is included here for full disclosure. Errors like this contribute to the peaks in the amplitude spectrum at the frame rate and higher harmonics. All original eye motion traces are available for download.

Six healthy subjects with normal vision were recruited for the study. Informed consent was obtained from each subject and all experimental procedures were reviewed and approved by the UC Berkeley Institutional Review Board and adhered to the tenets of the Declaration of Helsinki. To prepare subjects for AOSLO imaging, one drop of tropicamide (1%) and phenylephrine (2.5%) solution were administered topically 15 min prior to imaging to temporarily dilate the pupil and paralyze accommodation. For measurements of the motion amplitude spectrum in human eyes, AOSLO videos were recorded while subjects fixated a letter E optotype (Figure 1). The E was projected directly onto the retina at the center of the raster scan using the green channel (543 nm) in the AOSLO system while imaging was done with 840 nm near infrared (NIR) light. The combination of NIR light and very weak green background light (caused by light leaking through the acousto-optic modulator (AOM) that was used to project the letter E), formed a dim, reddish background over the extent of the raster scan. The E was 5 arcminutes in height, corresponding to a 20/20 letter. To keep the subject engaged in the fixation task over the course of each video, the E changed orientation to random positions at random time points ranging from 0.5 to 1.5 seconds. The subject was instructed to report its orientation every time it changed via the use of arrow keys on a keypad. Each subject completed five 30-s trials, for a total of 2.5 min of fixational eye motion per subject (with the exception of one subject, who only had four 30-s trials).

## Results

The purpose of the current study was to examine ocular tremor using the AOSLO system. The AOSLO system has advantages over other types of high-resolution eye tracking techniques due to its ability to image the retina directly, instead of having to infer retinal image motion from measurements taken from the anterior segment of the eye.

**Figure 2:**
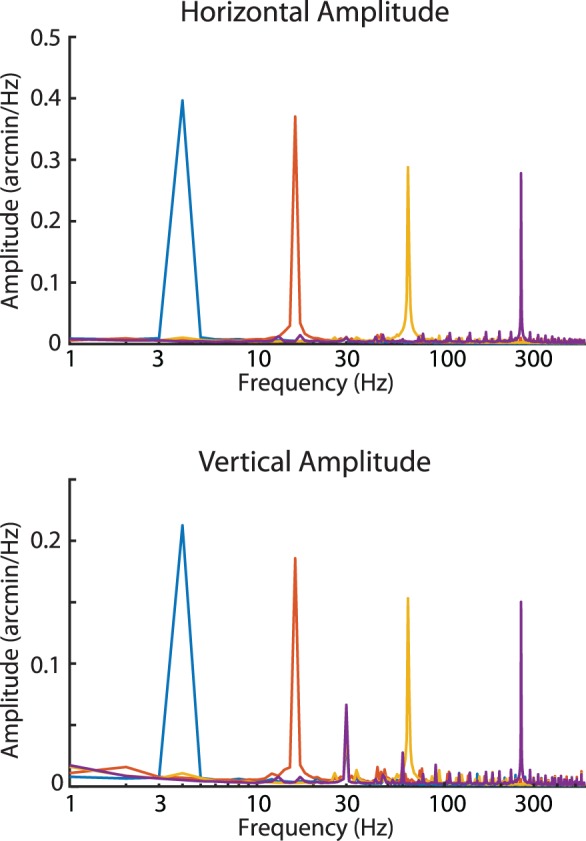
Amplitude spectra of motion from a moving model eye. Four videos were recorded from a model eye that was oscillated at four frequencies (indicated by the colors) with a fixed amplitude. For each of the input frequencies, the resultant amplitude spectra showed a peak of ∼0.4 arcminutes (horizontal) and just above ∼0.2 arcminutes (vertical) for each corresponding frequency. This simulation shows that we can recover high frequency, low amplitude motion with high fidelity from AOSLO videos.

Since the AOSLO system is a relatively novel eye tracker, the capabilities of the AOSLO system to measure small movements was validated in two ways. The first validation aimed to test the entire AOSLO system's ability to detect sinusoidal oscillations from a moving model eye. The resultant amplitude spectra plotted in Figure 2 showed a clear peak at the frequency of the sinusoidal oscillation for all input frequencies. Even though the motion of the sinusoidal oscillation was just a fraction of an arcminute, the AOSLO system was able to reliably recover the amplitude of the input motion. We suspect that the slight reduction in measured amplitude at the higher frequencies resulted from small relative shifts in the frequency between the galvanometer scanner or the AOSLO frame rate over the course of the video. Such shifts will cause slight distributions in amplitude to nearby frequencies in the spectrum. Human eye motion, which is broadband in frequency, will not be seriously affected by this limitation.

**Figure 3:**
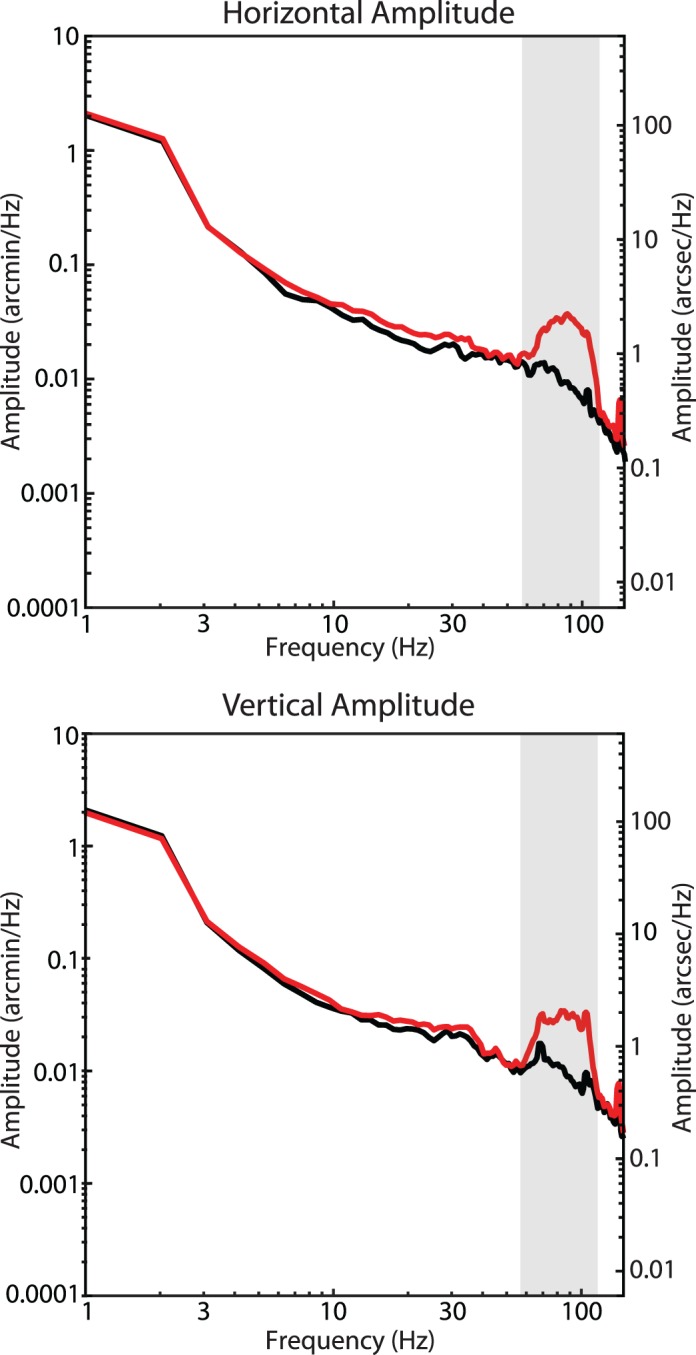
Amplitude spectra of an original AOSLO movie (black) and an AOSLO movie (red) that was digitally modified to include distortions from simulated tremor. The bump in the amplitude spectra from the modified AOSLO movie is clearly visible and matches the spectrum of the tremor that was digitally added.

The second validation was a simulation aimed to test the capabilities of the strip-based cross-correlation technique to recover motion from AOSLO movies. A real AOSLO movie was manipulated to add distortions consistent with a bandwidth of tremor-like motion between 50–100 Hz with a peak in the amplitude spectrum of ∼2 arcseconds. The distorted movie was then processed using the same strip-based cross correlation technique used on the human eye motion. The resultant amplitude spectrum of the manipulated movie compared to the original movie plotted in Figure 3 showed strong correlation except for a large bump in the amplitude spectrum between 50–100 Hz consistent with the input motion. Note that the amplitude spectra should theoretically match perfectly at all other frequencies, however the inclusion of the tremor-like signal subtly changed the samples which were flagged as saccades, so some small discrepancy between the two is to be expected. Regardless of this discrepancy, the strip-based cross-correlation technique was able to recover the tremor-like motion added to the modified movie. We compared the standard deviation of the retinal motion within the tremor band (50–100 Hz) with and without the artificially added tremor and found that the magnitude of motion added as expected. The standard deviation of the bandpass-filtered traces from the original movie was 4.8 arcseconds. The standard deviation of the tremor-like signal inserted into the movie was 10.9 arcseconds. The vector sum of the two components (square root of the sum of the squares) was 11.9 arcseconds, which matches up closely with the standard deviation of the modified movie (11.7 arcseconds).

**Figure 4:**
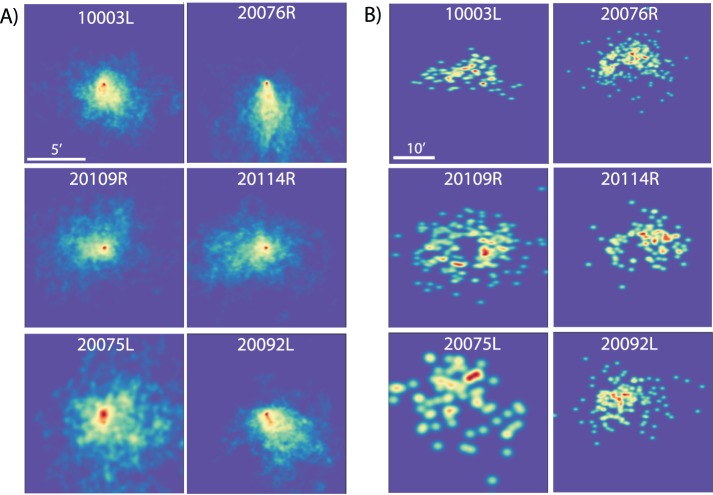
Heat maps of horizontal and vertical position for (A) drift and (B) saccades in space. All drift segments were offset to begin at 0,0 for display purposes, and the saccade heat maps show end points of each saccade relative to an origin of 0,0. Note that tendencies for some eyes to drift in specific directions are balanced by saccades in the opposite direction (e.g., 20076R). Scale bars of 5 and 10 arcminutes are shown on the top left panels of A and B, respectively. The color scale for each panel is normalized based on the amount of data available for that subject, with red indicating the highest frequency of occurrence.

Prior to performing the spectral analysis, basic metrics to describe the fixational eye motion were computed from the traces for each of the six subjects. Heat maps for saccade landing positions and drift segments are shown on Figure 4. All subjects showed normal fixational eye motion. Subjects made microsaccades roughly once per second (1.10 ± 0.57 microsaccades/s) with a normal amplitude (7.5 ± 1.5 arcminutes), speed (375 ± 49 arcminutes/s), and duration (36.9 ± 6.9 ms). Intersaccadic periods of drift also showed relatively normal statistics. Amplitude (3.8 ± 0.9 arcminutes); span, defined as the maximum distance from the mean location (3.2 ± 0.7 arcminutes); speed, defined as the mean of the instantaneous speed between each pair of samples within each drift segment (79.6 ± 15.6 arcminutes/second); and duration (620 ± 245 ms, min ∼33 ms, max 5.8 s) were all within normal parameters and were consistent with previous findings (Martinez-Conde, Macknik, & Hubel, 2004; Rucci & Poletti, 2015). All values are ±1 *SD*. Overall subjects did show slightly better fixational stability than average. This is likely due to a combination of the task (subjects had to attend to the orientation of a tumbling E) and the fact that all subjects were experienced in psychophysics experiments using the AOSLO system.

**Figure 5:**
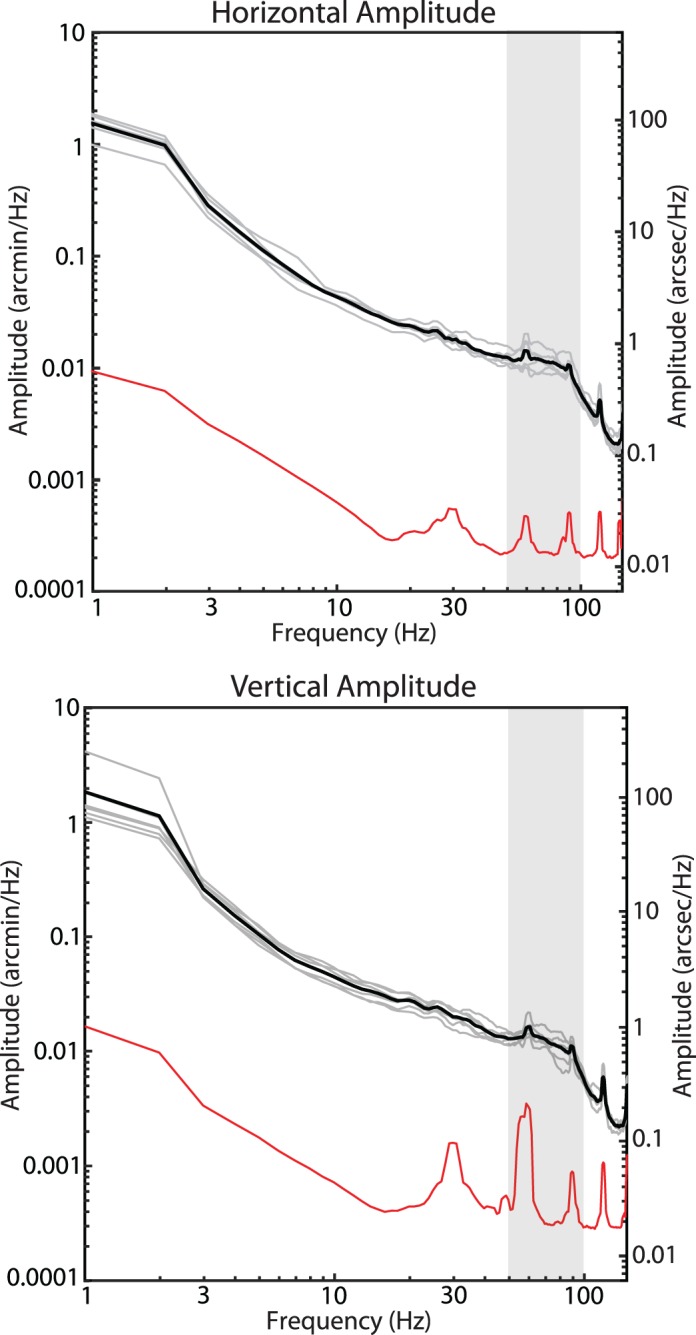
Amplitude spectra for six subjects and noise floor measured from a nonmoving model eye (red). Individual subjects' amplitude spectra are plotted in gray and the mean is plotted in black. The slight deviation from a linear 1/f falloff between 50–100 Hz (highlighted by the vertical gray bar) indicative of tremor is, on average, just over 1 arcsecond, much smaller than all previous reports of tremor. The spikes in the spectra are from periodic artifacts in the traces caused by tremor and residual reference frame distortions. The peaks are slightly broadened due to the multitaper spectral analysis method (Babadi & Brown, 2014). Note that the peaks appear larger in the noise floor, but this is due to the logarithmic scaling.

**Figure 6:**
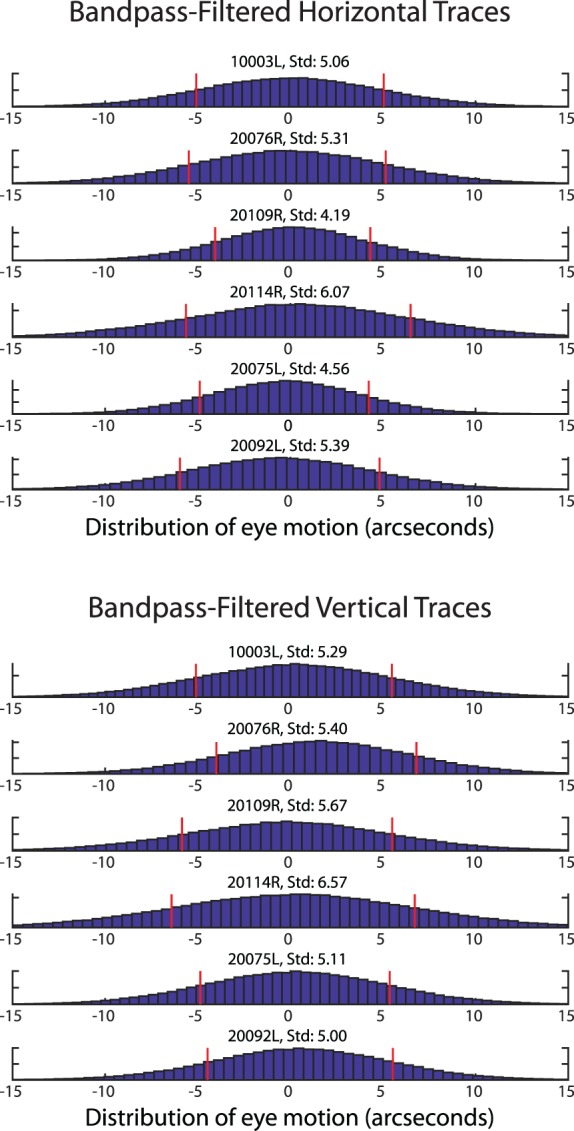
Histogram distribution of the bandpass-filtered drift traces. Each plot is a combination of all trials for each subject. The red stems indicate ±1 SD from the mean. All values are in seconds of arc.

Using the technique outlined in the methods, the amplitude spectra of fixational drift for each of the six subjects were calculated. The average horizontal and vertical amplitude spectra for all six subjects are shown in Figure 5. Similar to that reported in Ko et al. (2016), the spectra show a steeper than 1/*f* fall-off in amplitude but becomes 1/*f* after 10 Hz. However, unlike Ko et al. (2016) and others who used different tracking methods, the characteristic deviation from 1/*f* in the amplitude spectra indicative of tremor was very small. Although there was a slight elevation within the band of 50–100 Hz, the average amplitude of this deviation never exceeded 1 arcsecond and only one subject had an amplitude greater than 1 arcsecond within that range, much smaller than previous reports of tremor. To estimate the magnitude of motion on the retina caused by tremor, we bandpass-filtered all eye traces between 50 and 100 Hz and computed the standard deviation of the resulting traces. The distributions of the bandpass-filtered traces are shown in Figure 6. The standard deviation of the bandpass-filtered traces was 5.10 ± 0.66 arcseconds horizontally and 5.51 ± 0.57 arcseconds vertically. The standard deviation of the bandpass-filtered noise floor was 0.13 arcseconds horizontally and 0.55 arcseconds vertically, well below the measurements of the actual eye traces.

## Discussion

To assess the functional role that the tremor component of eye motion might have for human vision, its characteristics (amplitudes and frequencies) on the retinal image must be known. However, reports of the tremor in the literature vary widely and all published measurements are based on measurements made from the anterior segment, either from corneal reflections or a combination of reflections from the cornea and lens. In this paper, we present the first measurements of tremor on the retinal image directly employing the AOSLO as a retinal-image–based eye tracker.

As it is a new technology for eye tracking, we first validated that the AOSLO is capable of recording microscopic eye movements with high fidelity up to very high frequencies. The first validation used a moving model eye to test the AOSLO system's ability to precisely record eye motion from movies of high frequency and low amplitude. The second validation was a simulation where we used a digitally modified AOSLO movie to examine the capabilities of the offline analysis software to extract eye motion traces from movies of a real human retina as the subject engaged in an active fixation task. We then used the AOSLO to record fixational eye movements in six normal subjects to measure tremor on the retinal image directly while they engaged in an active fixation task, in this case reporting the orientation of a small rotating letter E optotype.

The temporal sampling rate of eye traces from the AOSLO system (1920 Hz) allows for analysis of frequencies up to 960 Hz, well beyond the 50–100 Hz bandwidth of tremor. The noise floor in the 50–100 Hz range measured from a non-moving model eye is <0.03 arcseconds in the amplitude spectrum, which is well below the amplitude of any eye motion previously reported, including tremor. The eye tracking capabilities of the AOSLO system, combined with direct retinal imaging, is therefore uniquely capable of analyzing the effects of small eye movements on the retinal image.

The measurements of retinal image motion from the six normal subjects during active fixation showed some evidence of tremor in the frequency range of 50–100 Hz, but it was very small with only one subject having an amplitude greater than 1 arcsecond at any point across that range. Even when there was evidence of tremor, the amplitude spectrum was monotonic (continuously declining) with increasing frequencies and was little more than a slight deviation from a 1/*f* curve. The resultant motion of the image on the retina caused by tremor was just over 5 arcseconds.

### Comparison with previously published results

We compared our measurements of tremor to three other reports in the literature wherein a similar spectral analysis of fixational eye motion was performed. With regard to the peak height of the tremor component in the amplitude spectrum, Ko et al. (2016) reported peak amplitudes of 4.8 and 3 arcseconds for the horizontal and vertical directions, respectively; Eizenman et al. (1985) reported a peak of 6 arcseconds and Findlay's (1971) plots show peaks ranging from 3 to 4 arcseconds. By comparison, the average height of the peak amplitude within the tremor band in our study was less than 1 arcsecond with only one subject out of the six having a slightly higher peak (Figure 5). It is important to note that the peak amplitude cannot be used directly to compute the magnitude of motion on the retina caused by tremor. To estimate the actual motion on the retina, we bandpass-filtered the eye motion traces to contain the tremor band only (50–100 Hz), computed the standard deviation of the resultant motion, and found the standard deviation of motion to be 5.10 ± 0.66 arcseconds horizontally and 5.51 ± 0.57 arcseconds vertically. Ko et al. (2016) did a similar analysis (their bandpass filter was between 40 and 80 Hz) and they found motions with standard deviations of 13.2 and 9 arcseconds for vertical and horizontal motion respectively. Neither Eizenman et al. (1985) nor Findlay (1971) did a bandpass-filtered eye motion analysis but, given the similarity between the spectra for those studies to Ko et al. (2016), we expect the motion caused by tremor to be similarly higher than ours.

Although there are some suggestions tremor could contribute to the visual percept through synchronization of retinal ganglion cells or through influencing the behavior of visual neurons in the brain (Greschner et al., 2002; Hennig et al., 2002), these studies generally assume the amplitude of tremor is around the scale of a foveal cone. Given that tremor on the retina is much lower than this, the possibility of this movement influencing the visual percept will need to be reexamined.

### Effects of cycloplegia

**Figure 7:**
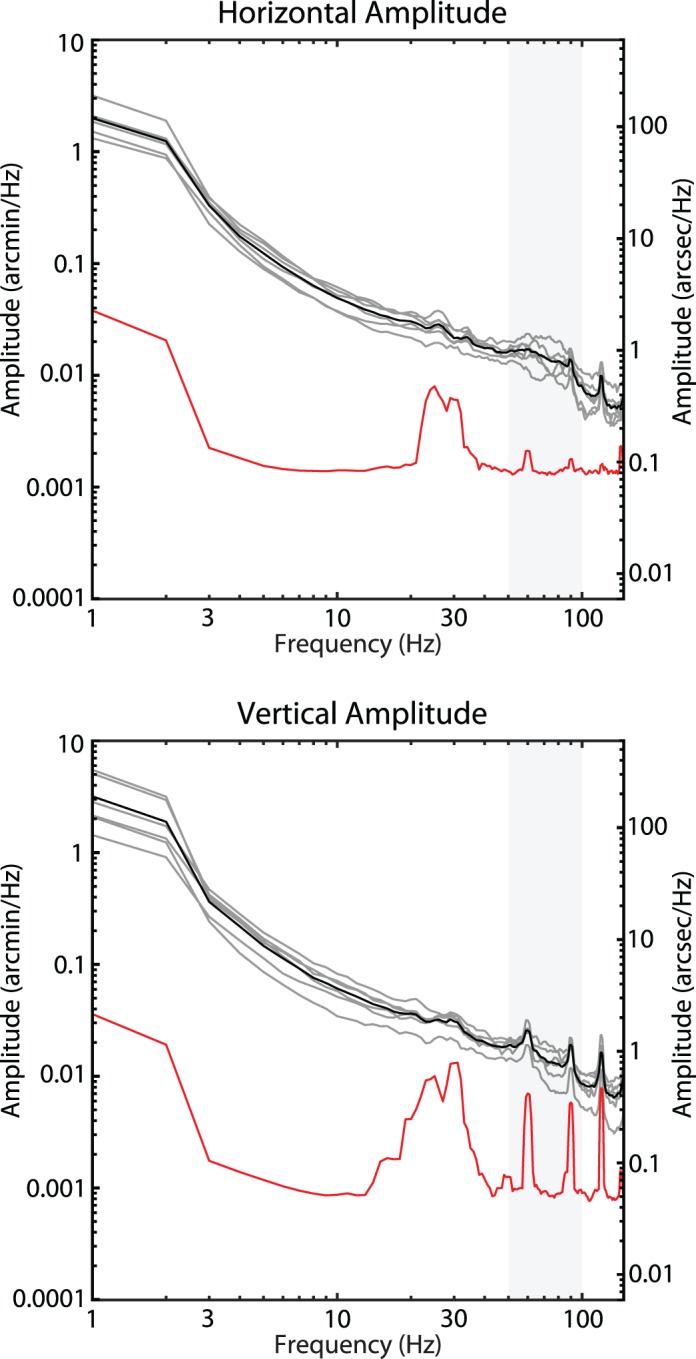
Amplitude spectra from fixational drift measured using the tracking scanning laser ophthalmoscope from six subjects without cycloplegia and noise floor measured from a nonmoving model eye (red). Individual subjects' amplitude spectra are plotted in gray and the mean is plotted in black. Eye motion with or without cycloplegia show a similar lack of any significant tremor on the retinal image.

In the current study, it was necessary to dilate and cycloplege subjects' eyes in order to achieve the best image quality for image-based eye tracking. Cycloplegia relaxes the ciliary muscle but, being that it is a sphincter muscle, it actually leads to an increase in the tension on the lens. Decreasing the tension on the lens is known to increase lens wobble (He, Donnelly, Stevenson, & Glasser, 2010). How this intervention might affect the magnitude and or presence of tremor is not well known. To address this question, we performed similar eye-tracking measurements in a tracking scanning laser ophthalmoscope (for details on that system, see Sheehy et al., 2012) for subjects that had not been cyclopleged. One 1-min video was collected for six subjects (four of whom also participated in the previous experiment) and the amplitude spectra were assessed using the same method described above. The results are shown in Figure 7. Compared to AOSLO, the spatial sampling resolution was ∼5 times lower (0.5 arcminutes per pixel) and the optical image resolution was worse. Nevertheless, the frequency bandwidth was the same and the noise floor was sufficient to detect tremor. Amplitude spectra measures without cycloplegia showed a similar small amplitude (<2 arcseconds) of tremor.

### Why are the current measurements lower than all previous reports?

At first glance, the negligible increase in the amplitude spectrum indicative of tremor on the retinal image is a surprising finding considering previous reports. Ultimately, tremor is not a well-defined concept and there appears to be large differences across subjects, eye tracking techniques, and quantitative analyses. Nevertheless, tremor has been consistently observed in eye motion traces obtained from a number of high-resolution eye tracking systems. It is important to note however, that all reports of tremor to date have relied on tracking eye motion from the anterior segment of the eye, and retinal image motion has only ever been inferred. The current study is based on unambiguous, high-resolution measurements of the retinal image motion directly.

In the following sections, we describe a combination of optical and biophysical factors that may serve to reduce the amplitude of tremor of the retinal image relative to entire eyeball. Some evidence indicating the presence of such reduction can be found in a report where AOSLO and DPI traces were recorded simultaneously (Stevenson & Roorda, 2005). In that report, eye motion traces from the two modalities were very similar except after microsaccades. However, no effort to compare estimates of tremor between the two tracking modalities was attempted in that paper. They speculated that overshoots caused by lens wobble that were detected in the DPI trace resulted in much smaller overshoots in movement of the retinal image as recorded in the AOSLO traces. Their experimental finding confirmed predictions made by Deubel and Bridgeman (1995).

There are two stages to modeling the differences between eye motion measured from the anterior segment and eye motion measured from retinal images. First is optical modeling and second is an analysis of the temporal dynamics of the lens.

### Optical modeling

**Figure 8:**
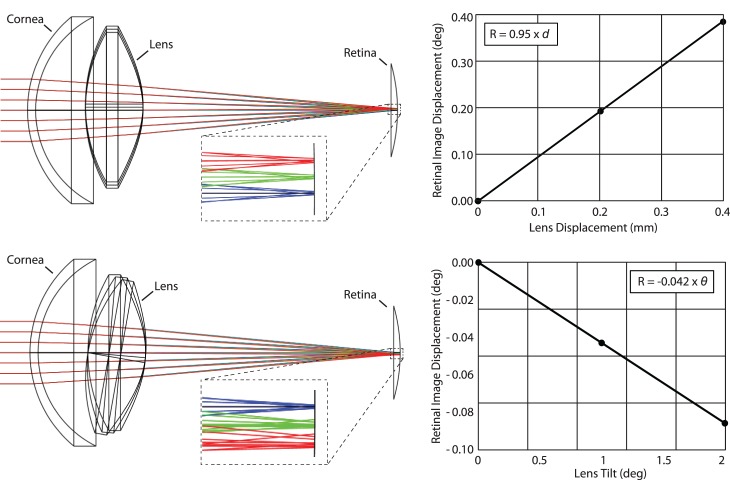
Zemax simulations. Top: Lens displacements of 0, 0.2, and 0.4 mm with their respective ray traces in blue, green, and red are illustrated in the drawing. A magnified inset is indicated by the dashed box. The focused spot is displaced in the same direction as lens displacement; 1 mm of lens displacement gives rise to just under 0.95° of displacement of the retinal image (assuming that 1° of visual angle corresponds to 300 microns on the retina). Bottom: Lens tilts of 0°, 1°, and 2° were tested (amplified tilts of 0°, 5°, and 10° with their respective ray traces in blue, green, and red are shown in the schematic and magnified inset to help to visualize the effect). In this case, the image displaces in the same angular direction as the tilt, but the effect is very small: 1° of tilt gives rise to −0.042° of retinal image displacement.

We used optical design software (Zemax, LLC, Kirkland WA) to model the effects of lens tilt and decentration in the schematic eye model available from the Zemax website (http://customers.zemax.com/os/resources/learn/knowledgebase/zemax-models-of-the-human-eye). Based on the optical model, we found that lens displacements and tilts both give rise to retinal image motion (Figure 8). The relationships between lens displacements and retinal image movement are:

\newcommand{\bialpha}{\boldsymbol{\alpha}}\newcommand{\bibeta}{\boldsymbol{\beta}}\newcommand{\bigamma}{\boldsymbol{\gamma}}\newcommand{\bidelta}{\boldsymbol{\delta}}\newcommand{\bivarepsilon}{\boldsymbol{\varepsilon}}\newcommand{\bizeta}{\boldsymbol{\zeta}}\newcommand{\bieta}{\boldsymbol{\eta}}\newcommand{\bitheta}{\boldsymbol{\theta}}\newcommand{\biiota}{\boldsymbol{\iota}}\newcommand{\bikappa}{\boldsymbol{\kappa}}\newcommand{\bilambda}{\boldsymbol{\lambda}}\newcommand{\bimu}{\boldsymbol{\mu}}\newcommand{\binu}{\boldsymbol{\nu}}\newcommand{\bixi}{\boldsymbol{\xi}}\newcommand{\biomicron}{\boldsymbol{\micron}}\newcommand{\bipi}{\boldsymbol{\pi}}\newcommand{\birho}{\boldsymbol{\rho}}\newcommand{\bisigma}{\boldsymbol{\sigma}}\newcommand{\bitau}{\boldsymbol{\tau}}\newcommand{\biupsilon}{\boldsymbol{\upsilon}}\newcommand{\biphi}{\boldsymbol{\phi}}\newcommand{\bichi}{\boldsymbol{\chi}}\newcommand{\bipsi}{\boldsymbol{\psi}}\newcommand{\biomega}{\boldsymbol{\omega}}\begin{equation}\tag{1}R = 0.95 * d\end{equation}

and

\newcommand{\bialpha}{\boldsymbol{\alpha}}\newcommand{\bibeta}{\boldsymbol{\beta}}\newcommand{\bigamma}{\boldsymbol{\gamma}}\newcommand{\bidelta}{\boldsymbol{\delta}}\newcommand{\bivarepsilon}{\boldsymbol{\varepsilon}}\newcommand{\bizeta}{\boldsymbol{\zeta}}\newcommand{\bieta}{\boldsymbol{\eta}}\newcommand{\bitheta}{\boldsymbol{\theta}}\newcommand{\biiota}{\boldsymbol{\iota}}\newcommand{\bikappa}{\boldsymbol{\kappa}}\newcommand{\bilambda}{\boldsymbol{\lambda}}\newcommand{\bimu}{\boldsymbol{\mu}}\newcommand{\binu}{\boldsymbol{\nu}}\newcommand{\bixi}{\boldsymbol{\xi}}\newcommand{\biomicron}{\boldsymbol{\micron}}\newcommand{\bipi}{\boldsymbol{\pi}}\newcommand{\birho}{\boldsymbol{\rho}}\newcommand{\bisigma}{\boldsymbol{\sigma}}\newcommand{\bitau}{\boldsymbol{\tau}}\newcommand{\biupsilon}{\boldsymbol{\upsilon}}\newcommand{\biphi}{\boldsymbol{\phi}}\newcommand{\bichi}{\boldsymbol{\chi}}\newcommand{\bipsi}{\boldsymbol{\psi}}\newcommand{\biomega}{\boldsymbol{\omega}}\begin{equation}\tag{2}R = 0.042 * \vartheta \end{equation}

where *R* is the retinal image displacement in degrees, *d* is the lens translation in mm, and ϑ is the lens tilt in degrees. Lens translations have the most profound effect: Tilting the lens also displaces the retinal image: the retinal image moves in the same direction as the movement of the optical axis of the lens, but the magnitude is negligible compared to displacement.

### Temporal dynamics of the lens

We considered how the lens might move within the tremoring eye, due to the fact that it is elastically supported by the zonules and ciliary body. The manner in which the crystalline lens moves in the eyeball following a saccade is a classic example of damped harmonic motion. He et al. (2010) confirmed this to be the case when they measured the motion of the lens following a saccade. A frequency analysis of one of the subjects in the study revealed the resonant frequency of lens oscillation to be about 20 Hz. The oscillation of the lens over time, *x*(*t*), dampens quickly, after about 1–2 cycles, following the equation:

\newcommand{\bialpha}{\boldsymbol{\alpha}}\newcommand{\bibeta}{\boldsymbol{\beta}}\newcommand{\bigamma}{\boldsymbol{\gamma}}\newcommand{\bidelta}{\boldsymbol{\delta}}\newcommand{\bivarepsilon}{\boldsymbol{\varepsilon}}\newcommand{\bizeta}{\boldsymbol{\zeta}}\newcommand{\bieta}{\boldsymbol{\eta}}\newcommand{\bitheta}{\boldsymbol{\theta}}\newcommand{\biiota}{\boldsymbol{\iota}}\newcommand{\bikappa}{\boldsymbol{\kappa}}\newcommand{\bilambda}{\boldsymbol{\lambda}}\newcommand{\bimu}{\boldsymbol{\mu}}\newcommand{\binu}{\boldsymbol{\nu}}\newcommand{\bixi}{\boldsymbol{\xi}}\newcommand{\biomicron}{\boldsymbol{\micron}}\newcommand{\bipi}{\boldsymbol{\pi}}\newcommand{\birho}{\boldsymbol{\rho}}\newcommand{\bisigma}{\boldsymbol{\sigma}}\newcommand{\bitau}{\boldsymbol{\tau}}\newcommand{\biupsilon}{\boldsymbol{\upsilon}}\newcommand{\biphi}{\boldsymbol{\phi}}\newcommand{\bichi}{\boldsymbol{\chi}}\newcommand{\bipsi}{\boldsymbol{\psi}}\newcommand{\biomega}{\boldsymbol{\omega}}\begin{equation}\tag{3}x\left( t \right) = a{e^{{\raise0.7ex\hbox{${ - \upsilon t}$} \!\mathord{\left/ {\vphantom {{ - \upsilon t} 2}}\right.\kern-.1pt}\!\lower0.7ex\hbox{$2$}}}}{\rm cos}\left( {{\omega _1}t - \phi } \right)\end{equation}

where *ω_1_* is the oscillating frequency, given by:

\newcommand{\bialpha}{\boldsymbol{\alpha}}\newcommand{\bibeta}{\boldsymbol{\beta}}\newcommand{\bigamma}{\boldsymbol{\gamma}}\newcommand{\bidelta}{\boldsymbol{\delta}}\newcommand{\bivarepsilon}{\boldsymbol{\varepsilon}}\newcommand{\bizeta}{\boldsymbol{\zeta}}\newcommand{\bieta}{\boldsymbol{\eta}}\newcommand{\bitheta}{\boldsymbol{\theta}}\newcommand{\biiota}{\boldsymbol{\iota}}\newcommand{\bikappa}{\boldsymbol{\kappa}}\newcommand{\bilambda}{\boldsymbol{\lambda}}\newcommand{\bimu}{\boldsymbol{\mu}}\newcommand{\binu}{\boldsymbol{\nu}}\newcommand{\bixi}{\boldsymbol{\xi}}\newcommand{\biomicron}{\boldsymbol{\micron}}\newcommand{\bipi}{\boldsymbol{\pi}}\newcommand{\birho}{\boldsymbol{\rho}}\newcommand{\bisigma}{\boldsymbol{\sigma}}\newcommand{\bitau}{\boldsymbol{\tau}}\newcommand{\biupsilon}{\boldsymbol{\upsilon}}\newcommand{\biphi}{\boldsymbol{\phi}}\newcommand{\bichi}{\boldsymbol{\chi}}\newcommand{\bipsi}{\boldsymbol{\psi}}\newcommand{\biomega}{\boldsymbol{\omega}}\begin{equation}\tag{4}{\omega _1} = {\left( {\omega _o^2 - {\raise0.7ex\hbox{${{\upsilon ^2}}$} \!\mathord{\left/ {\vphantom {{{\upsilon ^2}} 4}}\right.\kern-1.2pt}\!\lower0.7ex\hbox{$4$}}} \right)^{{\raise0.7ex\hbox{$1$} \!\mathord{\left/ {\vphantom {1 2}}\right.\kern-.1pt}\!\lower0.7ex\hbox{$2$}}}}\end{equation}

*ω_o_* is the resonant frequency, *a* is the amplitude, *ϕ* is the phase offset (not very important), and *υ* is a constant with dimensions of angular frequency indicating the strength of damping.

**Figure 9:**
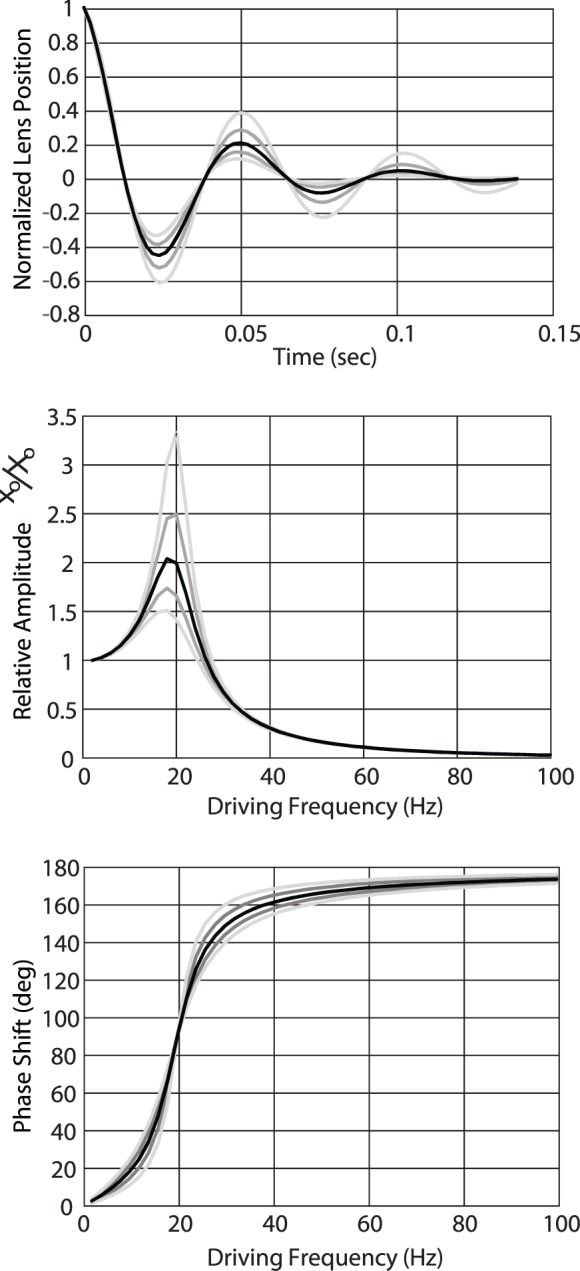
(A) Model of the position of a lens behaving as damped harmonic oscillator. In this case, the damping coefficient is 20π (10 Hz), or half of the resonant frequency of 20 Hz, which leads to about two cycles of oscillation before relaxing to zero. Gray shaded lines in all three plots show calculations with ±20% and ±40% changes in the damping coefficient. (B) Model of the amplitude of a lens behaving as a driven damped harmonic oscillator with resonant frequency of 20 Hz and range of damping coefficients. Driving frequencies near the resonant frequency gives rise to amplified motion of the lens. Driving frequencies within the range of tremor give rise to lens oscillations that are, on average, about 0.1 of the driving amplitude. Amplitudes of lens motion at the resonant frequency are highly dependent on the damping coefficient, but outside of that the trends are relatively similar. (C) Model of the phase shift of the lens motion. When driving frequencies are slow, the lens moves along with the eyeball as expected. For driving frequencies that are in the range of tremor (50–100 Hz) the lens moves in counter-phase with the eyeball. Changes in the damping coefficient have little effect on this trend.

Based on visual observation of the lens wobble artifact in He et al. (2010), we estimated the constant *υ* to be about half of the resonant frequency. A damped oscillation with a constant *υ = ω_o_/2* is plotted on Figure 9A and shows that the oscillation relaxes after one or two cycles.

The manner in which tremor affects the motion of the lens is a classic example of driven damped harmonic oscillation. The eyeball rotates about its center of rotation, and how the lens reacts to this motion depends on the damping constant *υ* and the resonant frequency *ω_o_* in the following way:

\newcommand{\bialpha}{\boldsymbol{\alpha}}\newcommand{\bibeta}{\boldsymbol{\beta}}\newcommand{\bigamma}{\boldsymbol{\gamma}}\newcommand{\bidelta}{\boldsymbol{\delta}}\newcommand{\bivarepsilon}{\boldsymbol{\varepsilon}}\newcommand{\bizeta}{\boldsymbol{\zeta}}\newcommand{\bieta}{\boldsymbol{\eta}}\newcommand{\bitheta}{\boldsymbol{\theta}}\newcommand{\biiota}{\boldsymbol{\iota}}\newcommand{\bikappa}{\boldsymbol{\kappa}}\newcommand{\bilambda}{\boldsymbol{\lambda}}\newcommand{\bimu}{\boldsymbol{\mu}}\newcommand{\binu}{\boldsymbol{\nu}}\newcommand{\bixi}{\boldsymbol{\xi}}\newcommand{\biomicron}{\boldsymbol{\micron}}\newcommand{\bipi}{\boldsymbol{\pi}}\newcommand{\birho}{\boldsymbol{\rho}}\newcommand{\bisigma}{\boldsymbol{\sigma}}\newcommand{\bitau}{\boldsymbol{\tau}}\newcommand{\biupsilon}{\boldsymbol{\upsilon}}\newcommand{\biphi}{\boldsymbol{\phi}}\newcommand{\bichi}{\boldsymbol{\chi}}\newcommand{\bipsi}{\boldsymbol{\psi}}\newcommand{\biomega}{\boldsymbol{\omega}}\begin{equation}\tag{5}{x_o} = {{\omega _o^2{X_o}} \over {{{\left[ {{{\left( {\omega _o^2 - {\omega ^2}} \right)}^2} + {\upsilon ^2}{\omega ^2}} \right]}^{{\raise0.7ex\hbox{$1$} \!\mathord{\left/ {\vphantom {1 2}}\right.\kern-.1pt}\!\lower0.7ex\hbox{$2$}}}}}}\end{equation}

and

\newcommand{\bialpha}{\boldsymbol{\alpha}}\newcommand{\bibeta}{\boldsymbol{\beta}}\newcommand{\bigamma}{\boldsymbol{\gamma}}\newcommand{\bidelta}{\boldsymbol{\delta}}\newcommand{\bivarepsilon}{\boldsymbol{\varepsilon}}\newcommand{\bizeta}{\boldsymbol{\zeta}}\newcommand{\bieta}{\boldsymbol{\eta}}\newcommand{\bitheta}{\boldsymbol{\theta}}\newcommand{\biiota}{\boldsymbol{\iota}}\newcommand{\bikappa}{\boldsymbol{\kappa}}\newcommand{\bilambda}{\boldsymbol{\lambda}}\newcommand{\bimu}{\boldsymbol{\mu}}\newcommand{\binu}{\boldsymbol{\nu}}\newcommand{\bixi}{\boldsymbol{\xi}}\newcommand{\biomicron}{\boldsymbol{\micron}}\newcommand{\bipi}{\boldsymbol{\pi}}\newcommand{\birho}{\boldsymbol{\rho}}\newcommand{\bisigma}{\boldsymbol{\sigma}}\newcommand{\bitau}{\boldsymbol{\tau}}\newcommand{\biupsilon}{\boldsymbol{\upsilon}}\newcommand{\biphi}{\boldsymbol{\phi}}\newcommand{\bichi}{\boldsymbol{\chi}}\newcommand{\bipsi}{\boldsymbol{\psi}}\newcommand{\biomega}{\boldsymbol{\omega}}\begin{equation}\tag{6}\phi = {{\rm tan}^{ - 1}}\left( {{{\upsilon \omega } \over {\omega _o^2 - {\omega ^2}}}} \right)\end{equation}

where *x_o_* is the amplitude of the oscillating mass (the lens), *X_o_* is the amplitude of the driver (eye tremor), *ω_o_* is the resonant frequency, *ω* is the frequency of the oscillating driving force (eye tremor), and *ϕ* is the phase shift between the driving oscillation and the oscillating mass.

Figure 9B and 9C plot the amplitude and phase of the lens oscillation that has a resonant frequency of *ω_o_ =* 40*π* (20 Hz) and a damping coefficient of *υ =* 0.5*ω_o_* over a range of driving frequencies. Note that for low frequencies, we see the expected behavior where the lens moves in phase with the eye with the same amplitude. When the driving frequency reaches the resonant frequency of the lens, the amplitude oscillation increases to about 2 times. At the same time, the phase of the lens movement transitions to counterphase motion. When the driving frequency increases further, the motion is in counterphase and the amplitude approaches zero. In the limit, the lens remains perfectly fixed in place relative to the tremoring eye. The modeled behavior remains qualitatively similar with variations in the damping constant *υ*, which were deduced from plots in He et al. (2010). Changes in the damping coefficient by up to 40% do not change the general trends in the plots. Similarly, changes in the resonant frequency will shift the curves but not in a manner that would alter the main conclusion, which is that during tremor, the lens moves with lower amplitude than the eyeball and it oscillates in counterphase to the eyeball.

**Figure 10:**
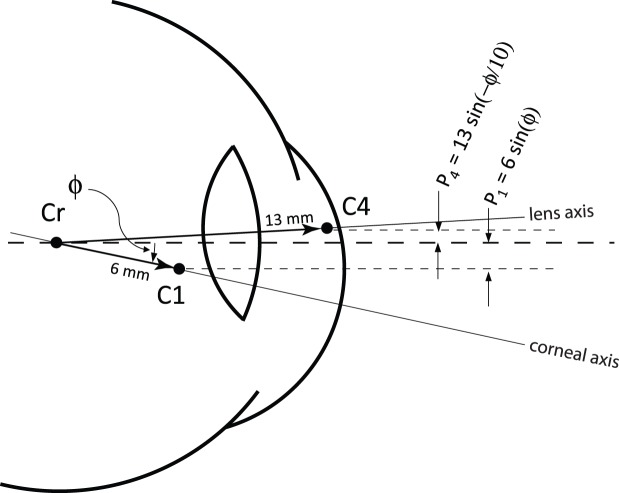
Schematic drawing showing how Purkinje reflections get displaced with rotations of the eye and lens. In the schematic, the eyeball has rotated downward by an angle ϕ and the lens has undergone a counterphase movement upward with 1/10 of the amplitude. For collimated incident light, the Purkinje reflections P1 and P4 are approximately positioned at the centers of curvature of their respective reflecting surfaces. The two reflections are displaced according to the equations on the figure. The total separation between P4 and P1 is 13_sin_(−ϕ/10) − 6_sin_(ϕ), which, for small angles, is −7.3 ϕ.

A counterphase motion of the lens relative to the eyeball would, in effect, amplify the motion that is estimated by the DPI eye tracker. The situation is illustrated schematically on Figure 10. In the DPI, the eye rotation is assumed to be proportional to the separation between the reflection from the cornea, P1, and the reflection from the back surface of the lens, P4, which are situated approximately at the center of curvatures of the surfaces that generate them (Cornsweet & Crane, 1973). If the lens and eyeball rotate together, as they would with slow rotations, then the separation between the two reflections (assuming small angles) is *∼*7*ϕ,* where *ϕ* is the rotation angle (in radians; Cornsweet & Crane, 1973). If the lens moves in counterphase to the eyeball rotation with 1/10 of the amplitude (as per estimations on Figure 8C), then the separation is ∼ *−*7.3 *ϕ*. The overall magnitude of the separation between the two reflections is 4.2% larger than the actual motion, and the sign is opposite.

The same lens translations that give rise to an overestimation of eye motion in the DPI would reduce the motion of the retinal image due to the prismatic effect illustrated in Figure 8. Consider an eyeball undergoing a tremor rotational motion with an amplitude of 1 arcminute. The amplitude of pupil displacement associated with the rotational eye movement is about 0.0029 mm (since the pupil is displaced about 10 mm from the center of rotation of the eye, giving rise to displacement of *S_P_ =* 10 sin (*ϕ*)).

The displacement of the lens relative to the pupil will move 0.0029 mm plus an additional 10% due to the counterphase oscillation (as indicated by the charts in Figure 9) for a total displacement of 0.0032 mm. According to Equation 1, these lens displacements will cause the amplitude of the retinal image motion to be smaller by 0.003° (0.18 arcminutes), or 82% of the eyeball rotation. In total, AOSLO measures of tremor amplitude should be 82% − 4.2% ≈ 78% of DPI measures.

However, tremor is not only detected in DPI traces, but has been seen in eye motion traces from search coil measurements as well as from traces of reflections from the cornea, neither of which involve a measurement of the lens. In these cases, assuming there is no artifact from the search coil or corneal reflection measurement, the only effect that will diminish the movement of the image on the retina from the overall eyeball motion is the translation of the lens which, according to the Zemax model, will give rise to a retinal motion amplitude that is 82% of the eyeball rotation.

Considering the arguments made above, the reported peak tremor amplitudes of 4.8 and 6 arcseconds from Ko et al. (2016) and Eizenman et al. (1985), respectively, would reduce to 3.75 and 4.92 arcseconds. Ko et al.'s (2016) average standard deviation of eye motion within the tremor band of 11.1 arseconds would reduce to 8.7 arcseconds. Our results remain lower than previous reports, even after correction, but they are in the same order of magnitude. The remaining differences could be due to the actual fixation task: our subjects were actively engaged in a fixation/acuity task, but Ko et al. (2016) had their subjects simply fixate the center of a blank screen and Eizenman et al. (1985). had their subjects fixate on a small source at 50 cm. Additionally, other biophysical factors, such as damping of lateral motion due to the elasticity of the retinal surface, could also be present. More experiments would be required to assess the effects of these possible causes for the differences.

### Conclusion

In this study, we measured the tremor component of eye motion by directly measuring the motion of the retina using the AOSLO eye tracker. We started by validating the AOSLO as a high-resolution, retinal-image–based eye-tracking technique that is uniquely able to resolve small movements of the retinal image caused by fixational eye movements. We first validated the capabilities of the AOSLO system by reliably recovering both the frequency and amplitude from recorded movies of an oscillating model eye. We were also able to recover a band of tremor that was artificially inserted into a real AOSLO movie. The AOSLO system was able to reliably measure the fixational eye motion of six human subjects as they participated in a tumbling E task. The statistics of both microsaccades and fixational drift were generally found to be within normal parameters of previous reports using high-resolution eye tracking. However, the amplitude of fixational tremor was smaller than all previous reports. Some, but not all, of the discrepancy is accounted for by optical and biomechanical factors that amplify measures of tremor-like eye motion based on measures from the anterior segment while damping the motion of the retinal image. Regardless of the cause, this paper shows that the amplitude of tremor in the eye during fixation is likely too small to have meaningful visual consequences.
